# ER Stress is Involved in Epithelial-To-Mesenchymal Transition of Alveolar Epithelial Cells Exposed to a Hypoxic Microenvironment

**DOI:** 10.3390/ijms20061299

**Published:** 2019-03-14

**Authors:** Eva Delbrel, Yurdagül Uzunhan, Abdoulaye Soumare, Thomas Gille, Dominique Marchant, Carole Planès, Emilie Boncoeur

**Affiliations:** 1 Université Paris 13, Sorbonne Paris Cité, Laboratoire ‘Hypoxie & Poumon’ (Inserm U1272), F-93017 Bobigny, France; eva.delbrel@univ-paris13.fr (E.D.); yurdagul.uzunhan@aphp.fr (Y.U.); abdoulaye.soumare@hotmail.com (A.S.); gille.tom@gmail.com (T.G.); dom.marchant@yahoo.fr (D.M.); carole.planes@aphp.fr (C.P.); 2 Assistance Publique Hôpitaux de Paris (APHP), Hôpital Avicenne, F-93017 Bobigny, France

**Keywords:** hypoxia, ER stress, alveolar epithelial cell, loss of phenotype

## Abstract

Background: Idiopathic pulmonary fibrosis (IPF) is a chronic, progressive and fatal interstitial lung disease of unknown origin. Alveolar epithelial cells (AECs) play an important role in the fibrotic process as they undergo sustained endoplasmic reticulum (ER) stress, and may acquire a mesenchymal phenotype through epithelial-to-mesenchymal transition (EMT), two phenomena that could be induced by localized alveolar hypoxia. Here we investigated the potential links between hypoxia, ER stress and EMT in AECs. Methods: ER stress and EMT markers were assessed by immunohistochemistry, western blot and qPCR analysis, both in vivo in rat lungs exposed to normoxia or hypoxia (equivalent to 8% O_2_) for 48 h, and in vitro in primary rat AECs exposed to normoxia or hypoxia (1.5% O_2_) for 2–6 days. Results: Hypoxia induced expression of mesenchymal markers, pro-EMT transcription factors, and the activation of ER stress markers both in vivo in rat lungs, and in vitro in AECs. In vitro, pharmacological inhibition of ER stress by 4-PBA limited hypoxia-induced EMT. Calcium chelation or hypoxia-inducible factor (HIF) inhibition also prevented EMT induction under hypoxic condition. Conclusions: Hypoxia and intracellular calcium are both involved in EMT induction of AECs, mainly through the activation of ER stress and HIF signaling pathways.

## 1. Introduction

Idiopathic pulmonary fibrosis (IPF) is a chronic, progressive and fatal interstitial lung disease of unknown origin. According to the prevailing hypothesis, IPF could be caused due to repetitive micro-injuries of the alveolar epithelium, followed by inefficient and/or aberrant epithelial repair and uncontrolled (myo)fibroblast proliferation, leading to excessive extracellular matrix deposition and destruction of distal lung architecture [1]. Indeed, in IPF lungs, some alveolar epithelial cells (AECs) become hyperplastic with abnormal activation and production of profibrotic factors such as TGF-β1, while other AECs undergo massive apoptosis. AECs may even acquire a mesenchymal phenotype through a process of epithelial-to-mesenchymal transition (EMT) [2,3,4]. In the last decade, chronic endoplasmic reticulum (ER) stress of AECs has been recognized as a key pathogenic event in lung fibrosis. ER stress is defined by the activation of the unfolded protein response (UPR) pathways to restore ER homeostasis [5,6]. 

This implies activation of the following transcription factors: activating transcription factor 4 (ATF4), ATF6N, and spliced X-box binding protein 1 (sXBP1), in order to inhibit protein translation, to increase chaperon synthesis, and to promote the ER activated degradation (ERAD) machinery. Altogether, ER stress and the UPR activation contribute to improve protein folding, and limit the formation of protein aggregates. First described in familial cases of lung fibrosis due to surfactant protein mutations, ER stress in AECs was also evidenced in sporadic cases of IPF [7,8]. We and others recently proposed that localized alveolar hypoxia could represent an important trigger for ER stress and subsequent UPR activation in AECs from sporadic IPF [9,10].

Localized alveolar hypoxia may likely occur in the course of IPF because of fibrotic lung remodeling and collapse of distal airspaces. Accordingly, the expression of the hypoxia-inducible factor 1α (HIF-1α) has been evidenced in AECs from IPF lungs as well as in AECs from mouse lungs treated with bleomycin, a drug known to induce pulmonary fibrosis [11,12]. We and others have reported in vitro, that hypoxic microenvironment induces phenotypic changes of primary AECs characterizing the EMT process. Indeed, HIF-1α can directly regulate EMT-inducing-genes through fixation on its hypoxia responsive element (HRE), but also promotes the pro-EMT transforming growth factor β (TGF-β) signaling pathways [13,14]. Furthermore, modulation of intracellular calcium has been reported in AECs exposed to hypoxia [15] and could be related to the induction of EMT features [16]. Hypoxic exposure may also lead to calcium depletion from the ER [15], thereby inducing ER stress. Although ER stress may promote EMT in some cell types [17], the potential involvement of chronic ER stress in hypoxia-induced EMT of AECs has never been studied. Here, we hypothesized that ER stress and activation of UPR pathways could contribute to EMT-associated phenotypic changes observed in AECs exposed to hypoxic stress, and that disturbance of intracellular calcium signaling could play a role in this process.

The objectives of the present study were therefore to examine the role of ER stress in the induction of EMT features in AECs exposed to hypoxia in vivo and in vitro, and to decipher the cellular mechanisms involved. We particularly studied the potential role of calcium, an important determinant of ER homeostasis. Our results provide evidence that ER stress and HIF-1 signaling pathways are both involved in the induction of EMT of AECs exposed to hypoxia, and suggest that any disturbance of intracellular calcium homeostasis plays a crucial role in this process.

## 2. Results

### 2.1. Acute Hypoxia Upregulates UPR Pathways and Induces Phenotypic Alterations of Alveolar Epithelial Cells in Rat Lungs

**Figure 1:**
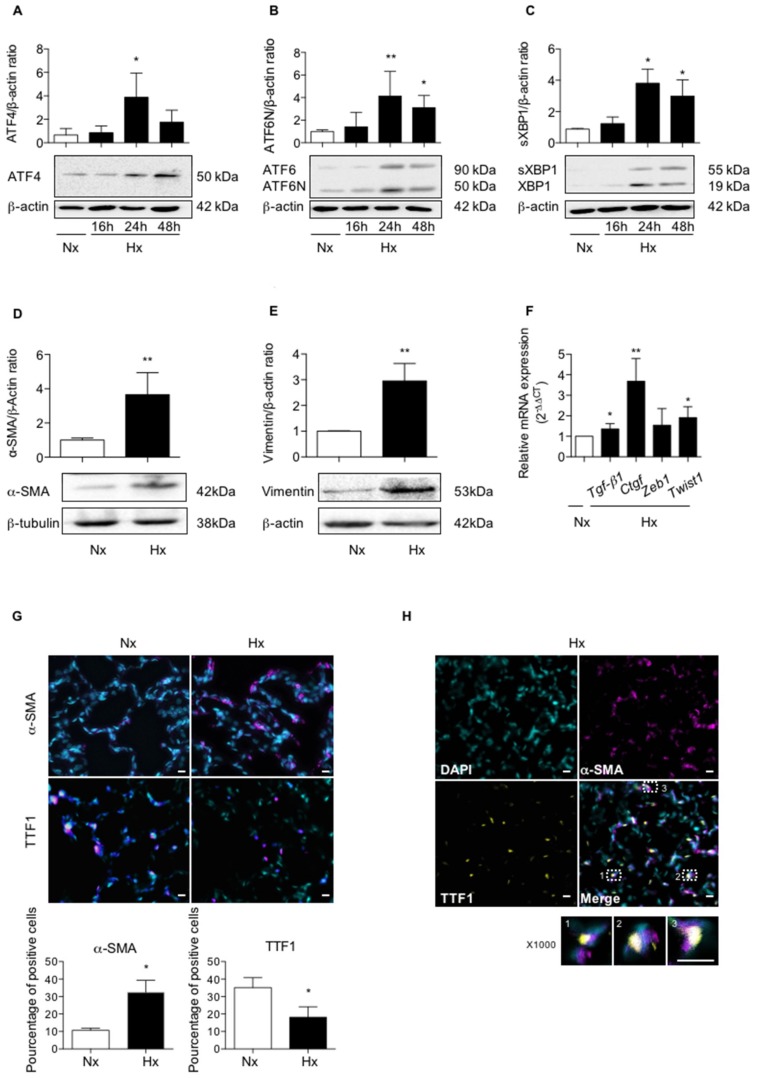
Hypoxia induces UPR pathways and modulates epithelial and mesenchymal markers expression in lungs of rats exposed to hypoxia. (**A**) Lungs of rats stabulated in normoxia (Nx) (21% O_2_) or exposed to hypoxia (Hx) (equivalent to 8% FiO_2_) during 16 h, 24 h, 48 h or 72 h were isolated and used for western blotting, immunohistochemistry and RT-qPCR analyses. Western blot of ATF4 protein, (**B**) ATF6N/ATF6 and (**C**) spliced XBP1 (sXBP1)/XBP1 were performed in lung homogenates. Representative blot of *n* = 8 experiments is shown. Quantification has been done and expression levels of ATF4, ATF6N or sXBP1 was reported to the β-actin expression level for each condition. Raw data were submitted to a Kruskal-Wallis one-way analysis of variance. * indicate a significant difference as compared with normoxic condition (*p* < 0.05). (**D**) Western blot of α-SMA protein and (**E**) vimentin were performed in lung homogenates from rat exposed to a 72 h-hypoxia. Representative blot of *n* = 5 experiments is shown. Quantification has been done and the expression of α-SMA (**D**) and vimentin was reported to the β-actin expression for each condition. Raw data (*n* = 5 rats in each group) were submitted to a Mann-Whitney test and relative expression was represented. (**F**) mRNA transcript expression levels of *Tgf-β1*, *Ctgf*, *Zeb1* and *Twist1* in lungs homogenates of rats exposed 48 h to hypoxia were quantified by qRT-PCR using 2^−∆∆CT^ method and reported to the normoxic condition. Raw data (*n* = 5 rats in each group) were submitted to a Mann-Whitney test and relative expression was represented. (**G**) Immunostaining of α-SMA or TTF1 were performed on 5 µm slices of paraffin-embedded from the left lobe of rat lung exposed to 72 h normoxia or hypoxia. A representative picture of at least *n* = 5 independent experiments for each condition has been presented. Original magnification: × 200 and scale bars represent 20 µm. Raw data were submitted to a Mann-Whitney test and relative expression was represented. (**H**) Co-staining for α-SMA (magenta), TTF1 (yellow), and DAPI to localize nuclei (in cyan) is shown (original magnification: × 400 or × 1000). Percentage of α-SMA or TTF1 positive cells in the lung of rats exposed 72 h to hypoxia as compared to normoxia (*n* = 5 rats in each group). Raw data were submitted to a Mann-Whitney test * and ** indicate a significant difference as compared with normoxic condition with a *p* < 0.05 and *p* < 0.01, respectively.

To evaluate the effect of hypoxia on UPR pathways in vivo, rats were exposed to a hypoxic atmosphere (equivalent to 8% FiO_2_) for 16 to 72 h. Compared to normoxia, exposure to hypoxia induced the three pathways of UPR signaling in lung tissues, as shown by western blot experiments (Figure 1A–C). A significant increase in ATF4 expression was observed after 24 h exposure (*p* < 0.05) as compared to normoxia, with no significant decrease at 48 h exposure (Figure 1A). To study the activation of the ATF6α pathway, the cleaved form of ATF6N (50 kDa) was quantified and the ATF6N/ATF6 ratio was compared in normoxic and hypoxic conditions. When normalized to β-actin, the exposure of rats to 24 h hypoxia led to a 4.2-fold increase of the ATF6N protein level compared to normoxia (Figure 1B), as well as an increase in the ATF6N/ATF6 ratio (2.8 ± 1.1, *p* < 0.05). This ATF6N expression was maintained at 48 h of hypoxic exposure, and a more than two-fold increase of the ATF6N/ATF6 ratio was still observed (2.08 ± 0.9). To study the activation of the XBP1 pathway, the spliced form of XBP1 (sXBP1: 55 kDa) was quantified, and sXBP1/XBP1 ratio was compared in normoxic and hypoxic conditions. When normalized to β-actin, exposure of rats to 24 h hypoxia led to a 3.8-fold increase of the sXBP1 protein level compared to normoxia (Figure 1C). This expression was maintained at 48 h of hypoxic exposure. However, no change in the sXBP1/XBP1 ratio was observed.

Expression of epithelial and mesenchymal markers was studied in parallel in lung homogenates of rat exposed to a 72 h-hypoxia. A 3-fold increase in the protein expression of α-SMA and vimentin was observed in hypoxic condition, as compared with normoxic condition (*p* < 0.01 and *p* < 0.01, respectively) (Figure 1D,E). In a previous work, our team demonstrated that hypoxia induces in vitro the expression of *Tgf-β1*, *Ctgf*, *Zeb1* and *Twist1* mRNA in AECs, four well-known genes involved in the process of EMT [18]. The effect of exposure to 48 h hypoxia on mRNA transcript expression levels of all these markers was therefore studied in rat lung homogenates (Figure 1F). As compared with normoxic condition, a significant increase in *Tgf-β1* expression, *Ctgf*, and *Twist1* was observed in hypoxic lungs (*p* < 0.05), while no significant modulation of *Zeb1* expression was observed in the hypoxic condition (Figure 1F). Finally, the expression and the localization of α-SMA and TTF1 (used as a marker of alveolar epithelial cells), were studied by immunofluorescence (Figure 1G,H). In hypoxic condition, co-immunostaining studies strongly suggest that α-SMA and TTF1 were co-expressed in some AECs (Figure 1H), consistent with an ongoing process of EMT.

### 2.2. Activation of UPR Pathways is Involved in the Loss of Alveolar Epithelial Cell Phenotype Induced in Vitro by Hypoxic Exposure

**Figure 2:**
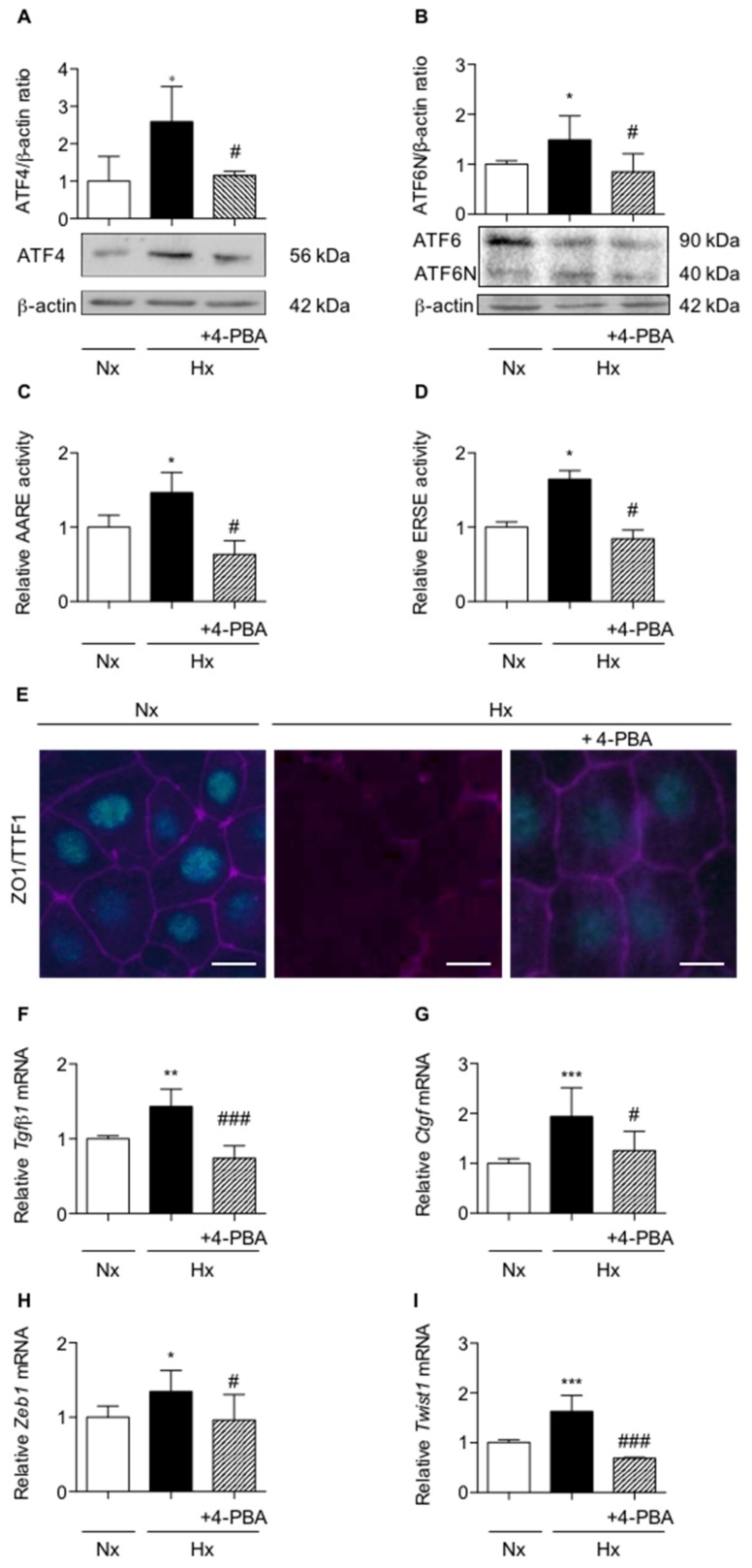
Inhibition of UPR pathways prevents loss of alveolar epithelial cells phenotype in primary rat alveolar epithelial cells exposed to hypoxia. Isolated primary rat AECs were cultured in normoxia (Nx) (21% O_2_) or hypoxia (Hx) (1.5% O_2_) during various periods in the presence or absence of 100 mM 4-phenylbutyrate (4-PBA). (**A**) Western blot of ATF4 protein and (**B**) ATF6N/ATF6 ratio were performed primary AECs exposed 6 h to hypoxia. Representative blot of *n* = 5 experiments is shown. Quantification has been done and the expression levels of ATF4 and ATF6 were reported to the β-actin expression for each condition. (**C**) Primary rat AECs transfected with plasmid coding for luciferase reporter activity of amino acid response element (AARE: i.e., ATF4-luc) or (**D**) endoplasmic reticulum stress element (ERSE: i.e., ATF6N/sXBP1-luc), were treated or not with 100 mM 4-PBA and exposed 6 h in hypoxic condition. (**C**) Luciferase activity corresponding to the transcriptional capacity of ATF4 or (**D**) ATF6N was measured. (**E**) ZO-1 (magenta) and TTF1 (cyan) immunostaining were performed on rat AECs cultured on filter and exposed for 6-days to hypoxia in the presence or absence of 100 mM 4-PBA. A representative picture of at least *n* = 5 independent experiments for each condition has been presented. Scale bar represents 50 µm. (**F**) mRNA expression levels of *Tgf-β1,* (**G**) *Ctgf*, (H) *Zeb1* and (I) *Twist1* were quantified by qRT-PCR using 2^−∆∆CT^ method in rat AECs cultured in the presence or absence of 100 mM 4-PBA and exposed 48 h to normoxia or hypoxia. mRNA levels under hypoxic condition were reported to the normoxic condition (*n* = 5 experiments). Raw data were submitted a Kruskal-Wallis test. *, ** and *** indicate a significant difference as compared with normoxic value with *p* < 0.05, *p* < 0.01 and *p* < 0.001 respectively. # and ### indicate a significant difference as compared with value in untreated hypoxic cells with *p* < 0.05 and *p* < 0.001, respectively.

Activation of UPR pathways was confirmed in vitro in primary rat AECs exposed for 6 h to hypoxia. As compared with normoxia, hypoxia led to a significant increase of ATF4 and ATF6N/ATF6 ratio protein expression (*p* < 0.01) (Figure 2A,B). Of note, we recently reported that the XBP1 spliced form was induced only after 16 h of hypoxic exposure [10]. In primary AECs transfected with plasmids coding for luciferase reporter activity of amino acid response element (ATF4-luc) or ER stress response element (ATF6N/sXBP1-luc), a significant induction of the luciferase activity was observed in response to hypoxia (*p* < 0.01) (Figure 2C,D). The ER stress inhibitor 4-phenylbutyrate (4-PBA), known as a chemical chaperone, limits UPR pathway activation. Indeed, treatment of AECs with 4-PBA fully prevented the increase in ATF4 protein expression and the ATF6N/ATF6 ratio (*p* < 0.05) (Figure 2A,B). Furthermore, treatment with 4-PBA also abolished the increase in ATF4 (Figure 2C) and ATF6N (Figure 2D) response element activities induced by hypoxia (*p* < 0.05).

We and others have previously demonstrated that a 6 day-exposure to hypoxia led to a change of AECs morphology, as illustrated by alteration of ZO-1 staining and the loss of TTF1 expression [18]. To investigate the implication of UPR pathways in this phenomenon, AECs were incubated with 4-PBA and cultured for 6 days under the hypoxic condition. As shown in Figure 2E, exposure of AECs to hypoxia led to a decrease in TTF1 expression and to modifications in cell morphology as assessed by ZO-1 staining. Although incubation of AECs with 4-PBA limited TTF1 and ZO-1 decreases, no significant modification in cell morphology was observed by ZO-1 staining. Then, *Tgf-β1*, *Ctgf*, *Zeb1* and *Twist1* mRNA levels were measured in rat primary AECs exposed for 48 h to hypoxia in the presence or absence of 4-PBA (Figure 2F–I). Treatment of AECs with 4-PBA fully prevented the hypoxia-induced increase in *Tgf-β1*, *Ctgf*, *Zeb1* and *Twist1* mRNA levels (*p* < 0.05) (Figure 2F–I).

### 2.3. Calcium Chelation Limits Cell Alterations and UPR Pathways Activation in AECs Exposed to Hypoxia

**Figure 3:**
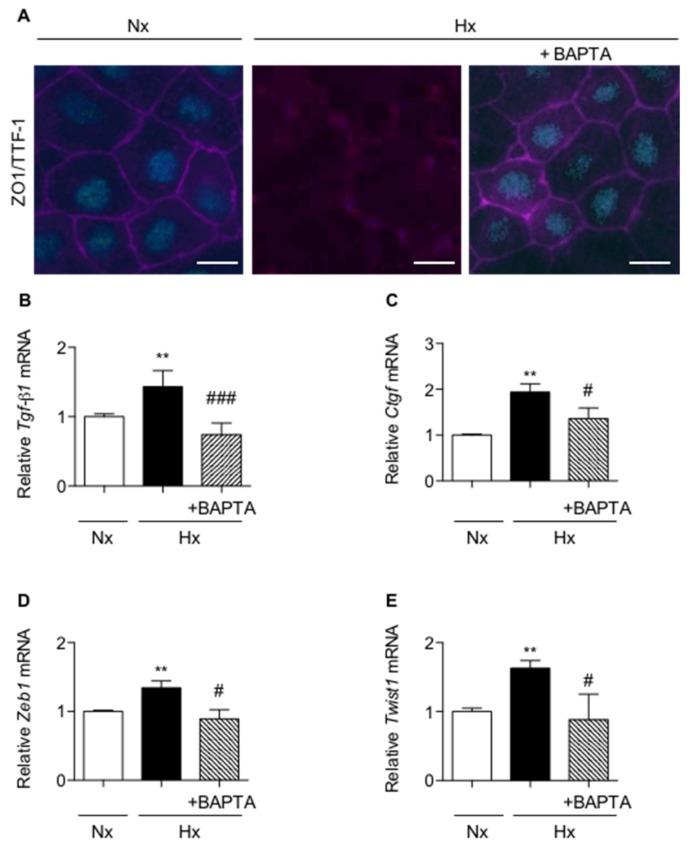
Calcium chelation prevents the loss of epithelial phenotype in primary rat alveolar epithelial cells exposed to hypoxia. Primary rat AECs were treated or not with 1 µM 1,2-bis(o-aminophenoxy)ethane-N,N,N,N-tetraacetic acid (BAPTA-AM) 90 min before exposure to normoxia (Nx) (21% O_2_) or hypoxia (Hx) (1.5% O_2_) for increasing times. (**A**) ZO-1 (magenta) and TTF1 (cyan) immunostainings were performed after a 6-day exposure. A representative picture of at least *n* = 4 independent experiments for each condition has been presented and scale bar represents 50 µm. (**B**) Expression levels of *Tgf-β1,* (**C**) *Ctgf*, (**D**) *Zeb1* and (**E**) *Twist1* mRNA transcripts were assessed after a 48 h exposure. mRNA levels were quantified by qRT-PCR using 2^−∆∆CT^ method and values in hypoxic cells were reported to values obtained in normoxic condition (*n* = 4 experiments). Raw data were submitted a Kruskal-Wallis test. ** indicate a significant difference as compared with normoxic condition with *p* < 0.01, respectively. # and ## indicate a significant difference as compared with value in untreated hypoxic cells with *p* < 0.05 and *p* < 0.01, respectively.

A fine tuning of calcium concentration is essential for ER homeostasis [19]. In AECs, hypoxia may induce calcium influx through L-type calcium channels located at the plasma membrane [20], as well as calcium depletion of the ER [15]. However, little is known about the role of intracellular calcium modulation on ER stress regulation. We first evaluated the involvement of calcium in hypoxia-induced loss of AECs phenotype. The cell permeable 1,2-bis(o-aminophenoxy)ethane-N,N,N,N-tetraacetic acid (BAPTA-AM) was used to chelate intracellular calcium. No change in cell morphology or phenotype was observed in normoxic-AECs treated with BAPTA-AM. Pre-treatment of AECs with BAPTA-AM just before a 6-day hypoxic exposure prevented the changes in cell morphology induced by hypoxia as illustrated by ZO-1 staining and the decrease in TTF1 expression (Figure 3A). The key role of calcium in the change of the AECs phenotype under hypoxia was reinforced by the observation that treatment with BAPTA-AM blunted the expression of *Tgf-β1*, *Ctgf*, *Zeb1* and *Twist1* mRNAs in hypoxic AECs (*p* < 0.05) (Figure 3B–E). 

**Figure 4:**
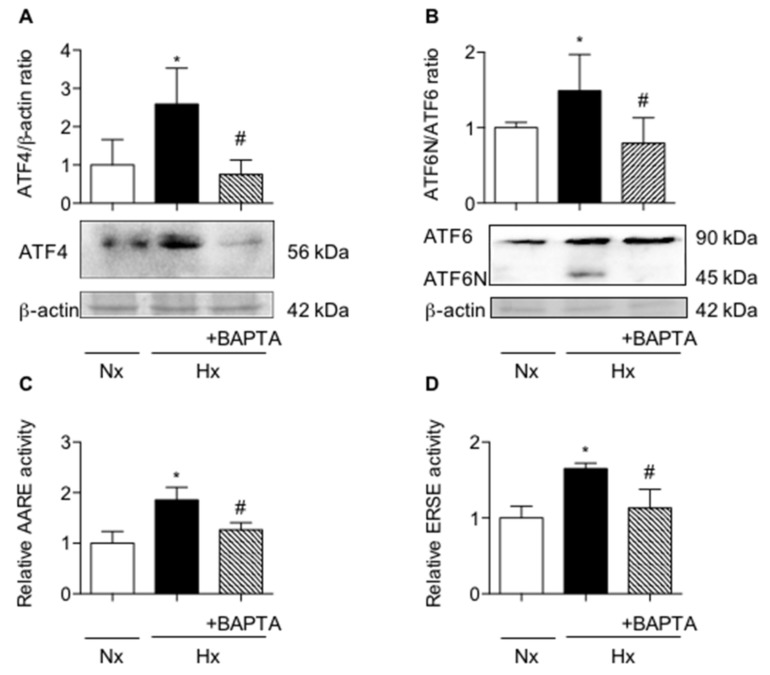
Calcium chelation prevents the induction of UPR pathways in primary rat alveolar epithelial cells exposed to hypoxia. Primary rat AECs were treated or not with 1 µM 1,2-bis(o-aminophenoxy)ethane-N,N,N,N-tetraacetic acid (BAPTA-AM) 90 min before a 6-h exposition to normoxia (Nx) (21% O_2_) or hypoxia (Hx) (1.5% O_2_). (**A**) Western blot of ATF4 and (**B**) ATF6N/ATF6 ratio were performed. Representative blot of *n* = 5 experiments is shown. Quantification of ATF4 and ATF6N protein expression was performed and was reported to the β-actin expression for each condition. (**C**) Primary rat AECs were transfected with plasmid coding for luciferase reporter activity of amino acid response element (AARE: i.e., ATF4-luc) or (**D**) endoplasmic reticulum stress element (ERSE: i.e., ATF6N/sXBP1-luc), and cultured as describe. (**C**) Luciferase activity corresponding to the transcriptional capacity of ATF4 or (**D**) ATF6N/sXBP1 was measured (*n* = 4 experiments). Raw data were submitted to a Kruskal-Wallis test. * indicates a significant difference against control or normoxic condition (*p* < 0.05). # indicates a significant difference as compared with value in untreated hypoxic cells (*p* < 0.05).

Furthermore, any expression and transcriptional activity of ATF4 and ATF6N/sXBP1 were fully prevented when AECs were pre-treated with BAPTA before exposure to hypoxia (Figure 4A–D, respectively, *p* < 0.05).

### 2.4. Pharmacological Inhibition of UPR Pathways and Chelation of Calcium Modulates HIF-1 Expression and Activity

**Figure 5:**
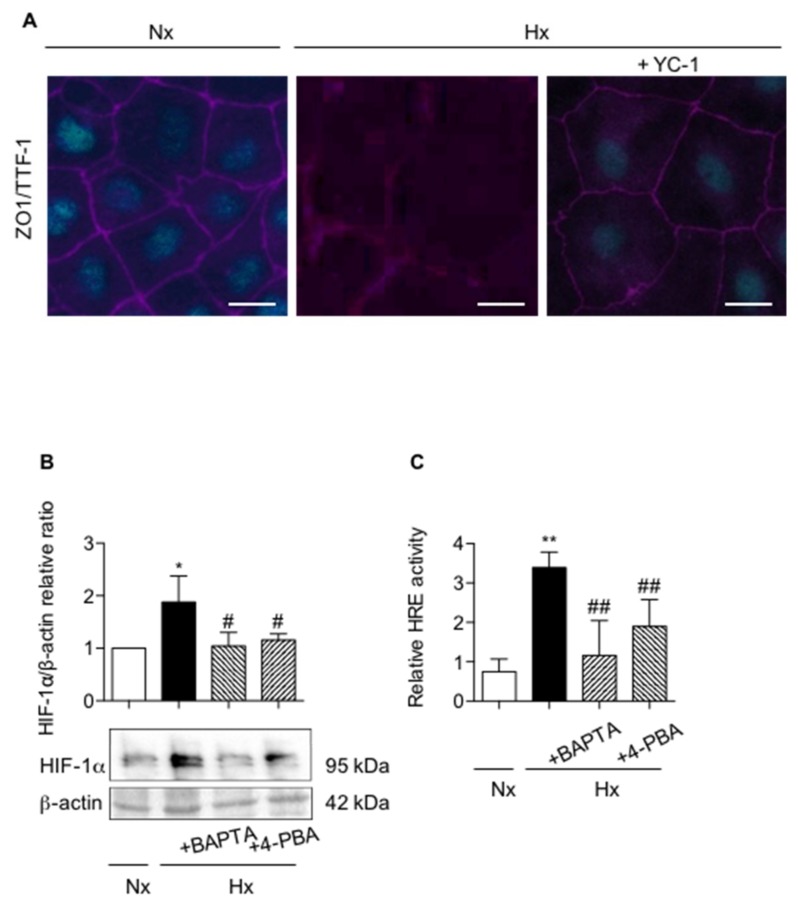
Involvement of HIF-1α in alveolar epithelial cell phenotypic changes induced by hypoxia. Primary rat AECs were cultured in normoxia (Nx) (21% O_2_) or hypoxia (Hx) (1.5% O_2_) during 6 days in presence or absence of 10 µM YC-1. (**A**) Immunostaining of ZO-1 (magenta) and TTF1 (cyan) were performed. *n* = 4 experiments were performed. Isolated primary rat AECs were cultured in normoxia (Nx) (21% O_2_) or hypoxia (Hx) (1.5% O_2_) during 6 h in the presence or absence of 100 mM 4-phenylbutyrate (4-PBA) or pre-treated or not with 1 µM BAPTA-AM 90 min before exposition to hypoxia. A representative picture of at least *n* = 4 independent experiments for each condition has been presented and scale bar represents 50 µm. (**B**) Western blot of HIF-1α protein levels was performed. Representative blot of *n* = 5 experiments is shown. Expression levels of HIF-1α were quantified and reported to β-actin expression for each condition. Primary rat AECs were transfected with plasmid coding for luciferase reporter activity of hypoxia responsive element (HRE: i.e., HIF-luc), and cultured as described. (**C**) Luciferase activity corresponding to the transcriptional capacity of HIF was reported (*n* = 4 experiments). Raw data were submitted a Kruskal-Wallis test. * and ** indicate a significant difference as compared with normoxic value with *p* < 0.05 and *p* < 0.01 respectively. # and ## indicate a significant difference as compared with value in untreated hypoxic cells with *p* < 0.05 and *p* < 0.01, respectively.

Numerous studies reported that HIF transcription factor could be involved in loss of AECs phenotype [21]. In our model, we showed by immunochemistry that treatment of AECs with the HIF-1 inhibitor YC-1 partially blunted the hypoxic effects on ZO-1 and TTF1 expression (Figure 5A). YC-1 limited TTF1 decrease in response to hypoxia, but no effect on cell morphology as assessed by ZO-1 expression was observed. As ER stress inhibition or calcium chelation also prevented the effect of hypoxia on ZO-1 and TTF1 expression, we next investigated the impact of UPR pathways inhibition or calcium chelation on HIF-1α stabilization and HIF capacity to transactivate the hypoxia responsive elements (HRE) upstream the luciferase gene in response to hypoxia (Figure 5B,C). In AECs exposed for 7 h to hypoxia, HIF-1α protein expression (Figure 5B) and the HRE activity (Figure 5C) were significantly increased as compared with normoxia (*p* < 0.05 and *p* < 0.01 respectively). Interestingly, treatment of AECs with either 4-PBA (to limit ER stress), or BAPTA-AM (to chelate calcium) during hypoxic exposure, completely suppressed the accumulation of HIF-1α protein (Figure 5B) and significantly blunted HIF transcriptional activity (Figure 5C) (*p* < 0.01), suggesting a link between ER stress, intracellular calcium concentration and the HIF pathway.

## 3. Discussion

Extracellular matrix remodeling and accumulation of fibroblasts and myofibroblasts is a hallmark of IPF. Epithelial-to-mesenchymal transition (EMT) of AECs has been increasingly documented in the fibrogenesis. EMT is mainly initiated by the presence of TGF-β, and we and others proposed that the hypoxic microenvironment of the cells could be an additive trigger for the EMT process in IPF [18,21]. In the present study, we provide evidence that hypoxic exposure and HIF induce EMT-like features in vivo and in vitro in AECs, and that ER stress and UPR-dependent signaling pathways are involved in this process.

Classically, loss of epithelial phenotype and acquisition of mesenchymal characteristics comprise upregulation of α-SMA and vimentin [4,21], modification of tight junction protein ZO-1 [4,17], and induction of transcription factors driving EMT such as *Zeb1* and *Twist1* [21]. In our study, prolonged exposure to hypoxia induced in vivo in rat lungs an overall increase in the expression of mesenchymal markers α-SMA and vimentin, of transcription factors driving EMT, and of pro-fibrotic mediators known to induce EMT. These in vivo results reinforce our previous in vitro work, demonstrating that prolonged exposure to hypoxia is a trigger for epithelial to mesenchymal modifications of AECs [21]. Furthermore, our results show increased expression of α-SMA in alveolar area in lung tissues from rats exposed to hypoxia. Co-immunostaining experiments suggest that the mesenchymal marker α-SMA and the epithelial marker TTF1 are co-expressed in some AECs, consistent with an ongoing process of EMT. Of note, co-expression of alveolar epithelial and mesenchymal markers has been observed previously in AECs from IPF lungs [17] and in bleomycin-induced lung fibrosis in the mouse [22]. Our observation favors the ambiguous role of AECs in the EMT phenomenon contributing to myofibroblasts accumulation in IPF, as previously described [2].

Our in vivo experiments also show that exposure to hypoxia induces the expression of ER stress signaling pathways, ATF4, ATF6N and sXBP1 with a specific time course in rat lung homogenates. Of note, the activation of XBP1 under hypoxia seems to be more delayed than the activation of ATF4 and ATF6α pathways. This result is consistent with previous studies suggesting that the ATF4/ATF6α branches of the UPR play an important role in early events such as apoptosis partly through C/EBP homologous protein (CHOP) induction, while XBP1 is mostly involved in the induction of EMT [10,23,24]. However we cannot exclude that part of the activation of XBP1 could be an implication in the induction of the late apoptosis [25].

Interestingly, in AECs, ER stress induction by thapsigargin or tunicamycin was shown to decrease epithelial markers E-cadherin and ZO-1, to increase the mesenchymal marker α-SMA, and to induce a fibroblast-like morphology consistent with EMT [17]. Furthermore, pharmacological inhibition of ER stress prevents TGF-β1-inducted EMT features [26]. Here, in line with *Mo* et al. [26] work, we observed that the incubation of primary rat AECs with the ER stress pharmacological inhibitor 4-PBA fully prevented the decrease in TTF1 expression induced by hypoxia, and the increase in EMT-like markers *Tgf-β1*, *Ctgf*, *Twist1* and *Zeb1*. However, no significant modification on cell morphology is observed through ZO-1 immunostaining. Interestingly, intracellular calcium chelation with BAPTA-AM had the same effect and prevented the loss of epithelial phenotype induced by hypoxic microenvironment. An important role of calcium as an intracellular messenger within this process has ever been proposed by others since inhibition of specific calcium channels or calcium chelation [16] in lung cancer cells limits TGF-β-induced EMT [27] though the limitation of N-cadherin and vimentin expression. Modulation of intracellular calcium level and ER homeostasis is largely documented. ER senses and integrates changes in calcium concentrations in and outside of the ER, and maintains cellular homeostasis through specific calcium dependent ER-associated proteins and the activation of the UPR pathways [28]. In our work, calcium chelation is able to limit hypoxia-induced ER stress and as a consequence to limit the EMT-like process. This observation is consistent with previous findings highlighting the impact of hypoxia on calcium homeostasis of AECs. Indeed, hypoxic exposure lead to an induction of calcium influx in cultured AECs, mostly through L-type calcium channels [20].

Next, we demonstrate the critical role of HIF-1α in the EMT process. Inhibition of HIF-1α stabilization by YC-1 prevented the loss of TTF1 expression. This observation is in line with studies conducted by Zhou et al. showing EMT features after HIF-1α stabilization in AECs exposed to hypoxia [21]. Interestingly, inhibition of HIF-1α was observed in hypoxic AECs treated either with the calcium chelator BAPTA, or with the ER stress inhibitor 4-PBA. These intriguing results may suggest that induction of EMT in AECs exposed to hypoxia results in a crosstalk between HIF-1α and ER stress signaling pathways. Interestingly, the induction of HIF-1 activity by ER stress has been previously reported in cardiomyocytes during myocardial ischemia-reperfusion [29]. Induction of HIF-1 could also be the result of an unbalance between pro-oxidative and anti-oxidative cellular responses. Interestingly, we previously demonstrated in vitro that hypoxic exposure increases reactive oxygen species (ROS) within AECs, and that this could be related to the activation of HIF-1 [30]. Here, we confirmed in vivo that exposure of rats to hypoxia increases ROS in the lungs (see Supplemental Figure S1A,B). As oxidizing environment is required for disulfide bonds formation within the ER, we tested the impact of antioxidant treatment of AECs with N-acetylcysteine (NAC) on the expression endoplasmic reticulum oxidoreductase 1 (ERO-1) involved in the disulfide bonds formation (Supplemental Figure S1C,D). 

We demonstrated ERO-1 induction in response to hypoxia, and showed that treatment of AECs with NAC limited this induction. However, the treatment with NAC did modify neither UPR activation (through the activation of ATF4 or ATF6N/sXBP1 responsive element) nor EMT induction (through the expression of ZO-1 and TTF1) in hypoxic cells (Supplemental Figure S1E,F and Figure 1G,H respectively). These findings suggest that the production of ROS does not play a prominent role in hypoxia-induced ER stress and related EMT.

**Figure 6:**
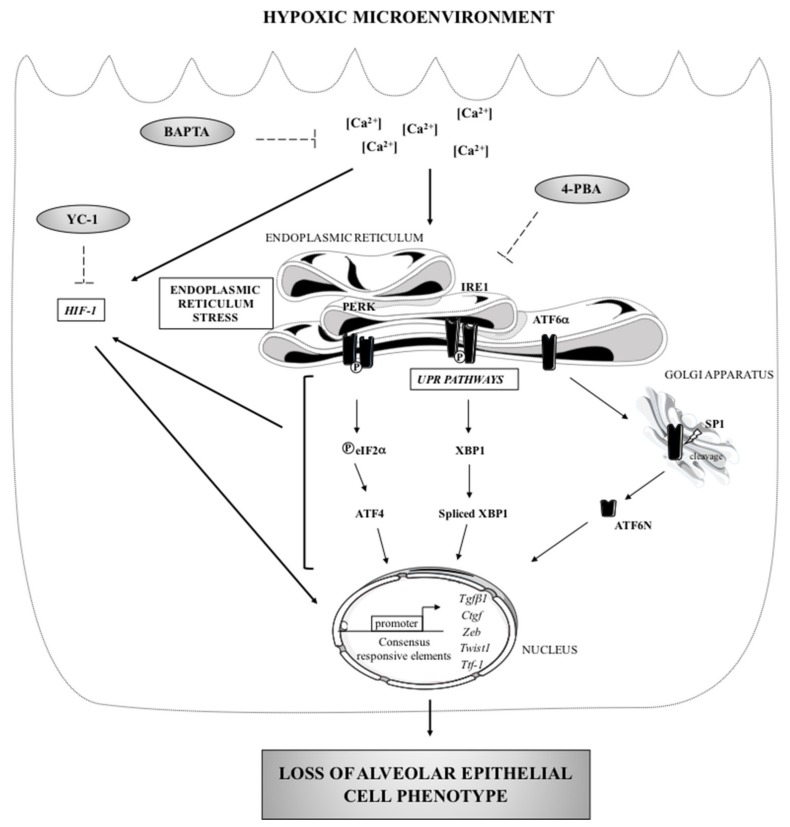
ER stress: a major regulator of the loss of epithelial cell phenotype in hypoxic environment. Loss of alveolar phenotype is a consequence of a long term exposure of the cells to a hypoxic microenvironment. Hypoxia leads to the activation of the PERK/ATF4 and ATF6α/ATF6N branches of the UPR, to the upregulation of *Tgfβ1*, *Ctgf*, *Zeb1* and *Twist1* gene expression, to a decrease in TTF1 expression, and finally to a disruption of ZO-1 expression (black arrows). In vitro, pharmacological inhibitors (dotted arrow) revealed that this effect is partly mediated by the stabilization of HIF-1α. Calcium chelation induced by BAPTA-AM prevents the loss of epithelial phenotype observed under hypoxia, by blunting the induction of the UPR and HIF-1 transcriptional activity.

In conclusion, the present study shows that hypoxia and the microenvironment of injured AECs can trigger epithelial cell dysfunction through induction of EMT features. Our study identifies the ER stress and the calcium present in the environment of the ER as critical elements governing the fate of injured cells. We provide evidence that inhibition of HIF-1α stabilization or pharmacological molecules that relieve ER stress can prevent hypoxia-induced alterations of AECs phenotype, at least in vitro (Figure 6). Taken together, and in accordance with recently published data from Burman et al. [9], our study strengthens the hypothesis that the hypoxic milieu of injured AECs could trigger lung fibrosis. Hypoxic-dependent ER stress signaling pathways could represent an attractive therapeutic approach to ameliorate epithelial abnormalities IPF.

## 4. Materials and Methods

### 4.1. Rat Model of Acute Hypoxic Exposure

Experiments have been approved by the local ethical committee (Charles Darwin, CE5) in accordance with the European Communities Council for animal care (C2EA-06, authorization APAFIS #7846, approved on September 2017, the 1st). 21-days old male Sprague-Dawley rats (Janvier Labs, St Berthevin, France) were housed four per cage and cared for a 12/12 light/dark cycle in a free food and water environment. Rats were placed for 16 h, 24 h, 48 h or 72 h (*n* = 6–8 rats per group) in a hypobaric chamber maintained at a pressure of 337 mmHg by a vacuum source at flow rates sufficient to prevent CO2 buildup to simulate an 8% FiO_2_. Control rats were kept outside of the hypobaric chamber in the same room. At the end of exposure, animals were anesthetized by intraperitoneal pentobarbital injection (60 mg/kg) and sacrificed by abdominal artery section. The left lobe of the lung was fixed by intratracheal instillation of fresh 4% paraformaldehyde (at 25 cm H_2_O transpulmonary pressure). The right lobe of the lung was snap frozen in liquid nitrogen.

### 4.2. Alveolar Epithelial Cells Isolation

Primary AECs were isolated from four weeks male Sprague-Dawley rats, as describe previously [21] according to a procedure approved by the local ethical committee (Charles Darwin, CE5) (C2EA-06, authorisation C9300801, authorization APAFIS #8150, approved on august 2017, the 8th). Briefly, perfused lungs were digested with 60 U elastase (Worthington, Serlabo, Entraigues-sur-la-Sorgue, France) and AECs were purified by a differential adherence technique on plastic plates (Greiner dishes, Fisher Scientific, Rungis, France). AECs were seeded at 4.5 × 10^6^ cells in 6-well plates or 1 × 10^6^ cells in 24-well plates containing polycarbonate membrane inserts with a pore size of 0.4 µm (Corning™ Transwell™, Fisher Scientific, Rungis, France). Cells were cultured in DMEM containing 25 mM D-glucose, 10 mM Hepes, 23.8 mM NaHCO3, 2 mM L-glutamine, 10% fetal bovine serum (FBS), 50 U/mL penicillin, 50 µg/mL streptomycin, 10 µg/mL gentamycin, 10 µg/mL amphotericin B (Thermo Fisher, Illkirch cedex, France) and placed at 37 °C with 5% CO_2_ in a humidified incubator.

### 4.3. Alveolar Epithelial Cells Treatments

On day 1 after isolation, primary rat AECs were placed in a humidified airtight incubator with inflow and outflow valves and exposed to a hypoxic gas mixture containing 1.5% O_2_-5% CO_2_-94.5% N_2_ (oxygen tension in culture media: 45 mmHg), and kept at 37 °C for six days. Control normoxic cells were maintained in a 21% O_2_-5% CO_2_-74% N_2_ humidified incubator during the same time (oxygen tension in culture media: 140 mmHg). For shorter hypoxic exposure experiments, primary rats AECs were used at day 3 after isolation. Calcium chelation was performed by a 90 min pre-treatment with 1 µM of the cell permeable 1,2-bis(o-aminophenoxy)ethane-N,N,N,N-tetraacetic acid (BAPTA-AM) (Sigma Aldrich, Saint Quentin Fallavier, France). Culture medium was removed, cells were washed with PBS and fresh medium was added before hypoxic exposure. 

To inhibit UPR pathways activation, AECs were treated with 100 mM of the ER stress inhibitor 4-phenylbutyric acid (4-PBA) (Sigma Aldrich, Saint Quentin Fallavier, France) and exposed for 6 h or 6-days hypoxia. To inhibit HIF-1α stabilization, AECs were treated with 10 µM 5′-hydroxymethyl-2′-furyl)-1-benzyl indazole (YC-1) and exposed for 6 h or 6-days hypoxia.

### 4.4. In Vivo and In Vitro Immunofluorescence Stainings

In vivo, after exposure for 72 h to hypoxia, fixed rat lungs were dehydrated, paraffin embedded, and sections of 5 µM were collected on classical Superfrost™ Plus microscope slides (Fisher Scientific, Rungis, France). Tissue sections were submitted to a pH 6 antigen retrieval, kept in a 2% BSA-5% goat serum blocking solution for 1 h at room temperature and incubated overnight at 4 °C with 1:500 anti-mouse alpha-smooth muscle actin (α-SMA) (A2547, Sigma Aldrich, Saint Quentin Fallavier, France) and 1:500 anti-rabbit thyroid transcription factor-1 (TTF1) solution antibodies (WRAB-1231, Seven hills, Cincinnati, OH, USA). Tissue sections were rinsed with PBS and incubated in 1:200 Alexa 568 Fluor^®^ conjugated secondary antibody solution (Invitrogen, Thermo Fisher, Illkirch cedex, France) and protected from light. In vitro, zonula occludens-1 (ZO-1) and TTF1 immunostaining were realized on AECs cultured 6 days in hypoxic condition as previously described [18]. Briefly, after 4% paraformaldehyde fixation and permeabilization with 0.1% Triton X100, AECs were incubated in 2% BSA blocking solution for 30 min and then 1 h with 1:200 anti-rabbit ZO-1 (AB2272, Sigma Aldrich, Saint Quentin Fallavier, France) and 1:50 anti-mouse TTF1 (180221, Invitrogen, Thermo Fisher, Illkirch cedex, France) antibodies solution. AECs were then washed with PBS and incubated in 1:200 Alexa 488 and 568 Fluor^®^ conjugated secondary antibodies solution (Invitrogen, Thermo Fisher, Illkirch cedex, France). For in vivo and in vitro immunofluorescence studies, nuclear labeling was performed by a DAPI (300 nM) staining during 8 min and washed with PBS. Slides were mounted with Immun-Mount solution (Thermo Fisher, Illkirch cedex, France) and imaged with a Retiga 2000R CCD camera with Image-proexpress 6.0 (QImaging, Briscous, France) connected to an Axio Observer D1 inverted epifluorescence microscope (Zeiss, Paris, France).

### 4.5. Transient Transfection of AECs and Luciferase Activity Assay

AECs were transiently transfected with the NEON™ transfection system (Life Technologies, Thermo Fisher, Illkirch cedex, France) allowing a 25–30% transfection efficiency as previously described [31]. ATF4 transcriptional activity was evaluated with the amino acid response element (AARE) cloned upstream of the firefly luciferase reporter gene (6.1 kb pGL3-2xAARE) [32]. ATF6N/sXBP1 transcriptional activity were evaluated with the endoplasmic reticulum response element (ERSE) also cloned upstream of the firefly luciferase reporter gene (pGL4.39 Promega, Charbonnières-les-Bains, France). Finally, HIF transactivation capacity was evaluated with a plasmid containing hypoxia response element (HRE) cloned upstream the luciferase reporter gene (pGL4.42 Promega, Charbonnières-les-Bains, France). The plasmid pRL-SV40 expressing renilla reniformis luciferase (RL) reporter gene (Promega, Charbonnières-les-Bains, France) was used for normalization of the luciferase response.

Freshly isolated AECs were plated at 1.5 × 10^7^ cells in 100 mm dishes. On day 1 after isolation, AECs were washed with PBS, dissociated with trypsin, centrifuged at 800 rpm during 8 min and resuspended in the resuspension buffer R (NEON, Invitrogen, Thermo Fisher, Illkirch cedex, France). In each tip, 4 × 10^5^ cells were mixed with 1 µg of the plasmid of interest (AARE-luc, ERSE-luc, HRE-luc) and with 0.4 µg of pRL-SV40. After electroporation, cells were seeded in 24-well plates without antibiotics in supplemented DMEM. On day 3, AECs were exposed to several treatments. At the end of exposition, AECs were lysed and Firefly and Renilla Luciferase assays were undertaken with the Dual-Luciferase Reporter Assay System, as specified by the manufacturer (Promega, Charbonnières-les-Bains, France). AARE, ERSE and HRE activities were assessed by quantifying the relative light units (RLU) ratio of firefly to RL measured with the Clarity™ luminescence microplate reader (BioTek, Colmar, France).

### 4.6. Western Blot Analyses

For in vivo study, 30 mg of snap-frozen rat lung biopsies exposed several times (16–48 h) to hypobaric hypoxia or normoxia were washed twice in PBS and then subjected to an Ultra-Turrax^®^ homogenizer (IKA, Staufen, Germany) in protein lysis buffer containing 20 mM Tris base, 150 mM NaCl, 1% Triton X100, 1% SDS, 0.5% deoxycholate and 0.01% cocktail protease inhibitors (Fisher Scientific, Rungis, France). 

For in vitro study, AECs exposed to normoxia or hypoxia were washed twice in ice-cold PBS and extracted in lysis buffer containing H_2_O, 62.5 mM Tris-HCl (pH 6.8), 10% Glycerol, 2% SDS and 0.01% cocktail protease inhibitors. Cells were submitted to three repeated cycles of sonication and speed freezing in azote. Cell lysates were then centrifuged at 13,000 rpm for 30 min at 4 °C to eliminate debris. Total protein content in lung homogenates or in AECs was determined by BCA assay (Thermo Fisher, Illkirch cedex, France). Samples (30 µg protein per sample in Laemmli buffer containing 5% β-mercapto-ethanol) were then resolved through 7.5% or 12% acrylamide gels, electrically transferred to nitrocellulose membrane, and incubated overnight at 4 °C with 1:2000 anti-mouse ATF4 (sc-200, Santa Cruz Biotechnology, Cliniscience, Nanterre, France) anti-mouse α-SMA (A2547, Sigma Aldrich, Saint Quentin Fallavier, France), anti-mouse vimentin (V6630, Sigma Aldrich, Saint Quentin Fallavier, France) or with 1:500 anti-rabbit ATF6α (sc-22799, Santa Cruz Biotechnology, Cliniscience, Nanterre, France), anti-mouse XBP1 (sc-7160, Santa Cruz Biotechnology, Nanterre, France), anti-rabbit HIF-1α (NB100-479, Novus, Biotechne, Lille, France) antibody solution. Next day, the membrane was incubated with appropriate anti-rabbit or mouse HRP-conjugated secondary antibody (Dako, P0448 and P0447 respectively, DAKO, Agilent technologies, Les Ulis Cedex, France). The signals revealed after membrane incubation in SuperSignal™ chemiluminescent HRP substrates (Thermo Fisher, Illkirch cedex, France) were quantified by densitometric analysis using Image Lab™ software (BioRad, Marnes-la-Coquette, France), normalized using quantification of the β-actin or β-tubulin in each lane and expressed in arbitrary units.

### 4.7. RNA Extraction and Reverse Transcription-Quantitative Polymerase Chain Reaction

AECs freshly isolated were seeded at 3.5 × 10^6^ cells on 12-well plates. After normoxia or hypoxia and treatments exposition, cells were washed twice with PBS, extracted in ice-cold PBS and centrifuged 5 min at 1500 rpm. For in vivo study, 30 mg of snap-frozen rat lung biopsies exposed 48 h to hypobaric hypoxia or normoxia were washed twice in PBS. TRIzol™ Reagent (Qiagen, Courtaboeuf, France) was added and lung tissue was homogenized with an Ultra-Turrax^®^ system. AECs pellet was also incubated with TRIzol™ Reagent. After vigorous mixing cell and lung homogenates, chloroform was added and centrifuged 15 min at 12,000 g. After transferring aqueous phase, nucleic acids were precipitated by isopropanol and then by 75% ethanol. Extracted ARN were resuspended in 20 µL H_2_O and quantified using a BioSpec-Nano spectrophotometer (Life Science, Shimadzu, Marne-la-Vallée, France) at 260 nm. Single-strand cDNAs were synthesized from 0.5 age of total RNA using maxima first strand cDNA synthesis kit composed by a mixture of oligo (dT) and random hexamer primers according to the manufacturer’s instructions (Thermo Fisher, Illkirch cedex, France). Resulting cDNA samples were amplified by quantitative polymerase chain reaction (PCR) with Absolute qPCR SybrGreen Rox mix (Thermo Fisher, Illkirch cedex, France) on StepOne Plus Real-Time PCR system (Applied Biosystem, Life technology, Thermo Fisher, Illkirch cedex, France). Loss of epithelial phenotype was evaluated by measurement of *Tgf-β1*, *Ctgf*, *Twist1* and *Zeb1* transcripts levels. Length and sequence of primers used for quantitative real-time PCR are described in Table 1. All samples were performed in duplicate. Cycle threshold values were normalized to the amplification of the endogenous reference gene β-actin for each transcript. The expression levels were calculated using the 2^−∆∆CT^ method and fold increase expression were reported to normoxia condition.

### 4.8. Statistical Analyses

Results were presented as means ± SD. To evaluate differences between groups, all raw data were submitted to a Mann-Whitney and Kruskal-Wallis one-way analysis of variance followed by a Dunn’s multiple comparison tests and graphics were performed by PRISM software (version 6, GraphPad, San Diego, CA, USA). A *p* value < 0.05 was considered a significant difference between conditions.

## Figures and Tables

**Table 1:** Primers used for Real-Time Polymerase Chain Reaction.

GENE	Forward Primer	Reverse Primer
*Twist1* (108 bp)	5′-CTACGCCTTCTCCGTCTGGA-3′	5′-CAATGACATCTAGGTCTCCGGC-3′
*Ctgf* (100 bp)	5′-CCTAGCTGCCTACCGACTGG-3′	5′-CTTAGAACAGGCGCTCCACT-3′
*Tgf-β1* (146 bp)	5′-TGAGTGGCTGTCTTTTGACG-3′	5′-TGGGACTGATCCCATTGATT-3′
*Zeb1* (90 bp)	5′-CCGTAAGTTCAAGTGCACCG-3′	5′-GTGGGACTGCCACTGTGGAT-3′
*β-actin* (74 bp)	5′-ACCGTGAAAAGATGACCCAGA-3′	5′-CACAGCCTGGATGGCTACGT-3′

## Supplementary Materials

Supplementary materials can be found at https://www.mdpi.com/1422-0067/20/6/1299/s1.

## Abbreviations

4-PBA	4-phenylbutyrate
AARE	Amino-acid responsive element
AEC	Alveolar epithelial cell
ATF	Activating transcription factor
BAPTA	1,2-bis(o-aminophenoxy)ethane-N,N,N,N-tetraacetic acid
ER	Endoplasmic reticulum
ERSE	ER stress element
HIF	Hypoxia inducible factor
HRE	Hypoxia responsive element
IPF	Idiopathic pulmonary fibrosis
IRE-1	Inositol-requiring endonuclaease-1
mRNA	messenger RNA
PERK	PKR-ER like kinase
RL	Renilla reniformis luciferase
TTF1	Thyroid transcription factor 1
UPR	Unfolded protein response
YC-1	5′-hydroxymethyl-2′-furyl)-1-benzyl indazole
ZO-1	Zonula occludens 1

## References

[1] Selman M., King T.E., Pardo A., American Thoracic Society, European Respiratory Society, American College of Chest Physicians (2001). Idiopathic Pulmonary Fibrosis: Prevailing and Evolving Hypotheses about Its Pathogenesis and Implications for Therapy. Ann. Intern. Med..

[2] Goldmann T., Zissel G., Watz H., Drömann D., Reck M., Kugler C., Rabe K.F., Marwitz S. (2018). Human Alveolar Epithelial Cells Type II Are Capable of TGFβ-Dependent Epithelial-Mesenchymal-Transition and Collagen-Synthesis. Respir. Res..

[3] Kugler K., Matthias C., Wolters P.J., Robillard L., Galvez M.G., Brumwell A.N., Sheppard D., Chapman H.A. (2006). Alveolar Epithelial Cell Mesenchymal Transition Develops in Vivo during Pulmonary Fibrosis and Is Regulated by the Extracellular Matrix. Proc. Natl. Acad. Sci. USA.

[4] Willis B.C., Liebler J.M., Luby-Phelps K., Nicholson A.G., Crandall E.D., du Bois R.M., Borok Z. (2005). Induction of Epithelial-Mesenchymal Transition in Alveolar Epithelial Cells by Transforming Growth Factor-Beta1: Potential Role in Idiopathic Pulmonary Fibrosis. Am. J. Pathol..

[5] Gora S., Maouche S., Atout R., Wanherdrick K., Lambeau G., Cambien F., Ninio E., Karabina S.-A. (2010). Phospholipolyzed LDL Induces an Inflammatory Response in Endothelial Cells through Endoplasmic Reticulum Stress Signaling. FASEB J..

[6] Guleria A., Singh V., Chandna S. (2017). An Attenuated Calcium Signaling and Pre-Emptive Activation of UPR Pathway Together Contribute to ER and Calcium Stress Resilience of Lepidopteran Insect Cells. Biochim. Biophys. Acta (BBA) Gen. Subj..

[7] Korfei M., Ruppert C., Mahavadi P., Henneke I., Markart P., Koch M., Lang G., Fink L., Bohle R.-M., Seeger W. (2008). Epithelial Endoplasmic Reticulum Stress and Apoptosis in Sporadic Idiopathic Pulmonary Fibrosis. Am. J. Respir. Crit. Care Med..

[8] Lawson W.E., Crossno P.F., Polosukhin V.V., Roldan J., Cheng D.-S., Lane K.B., Blackwell T.R., Xu C., Markin C., Ware L.B. (2008). Endoplasmic Reticulum Stress in Alveolar Epithelial Cells Is Prominent in IPF: Association with Altered Surfactant Protein Processing and Herpesvirus Infection. Am. J. Physiol. Lung Cell. Mol. Physiol..

[9] Burman A., Kropski J.A., Calvi C.L., Serezani A.P., Pascoalino B.D., Han W., Sherrill T., Gleaves L., Lawson W.E., Young L.R. (2018). Localized Hypoxia Links ER Stress to Lung Fibrosis through Induction of C/EBP Homologous Protein. JCI Insight.

[10] Delbrel E., Soumare A., Naguez A., Label R., Bernard O., Bruhat A., Fafournoux P., Tremblais G., Marchant D., Gille T. (2018). HIF-1α Triggers ER Stress and CHOP-Mediated Apoptosis in Alveolar Epithelial Cells, a Key Event in Pulmonary Fibrosis. Sci. Rep..

[11] Guo L., Xu J., Liu L., Liu S., Zhu R. (2015). Hypoxia-Induced Epithelial-Mesenchymal Transition Is Involved in Bleomycin-Induced Lung Fibrosis. BioMed Res. Int..

[12] Tzouvelekis A., Harokopos V., Paparountas T., Oikonomou N., Chatziioannou A., Vilaras G., Tsiambas E., Karameris A., Bouros D., Aidinis V. (2007). Comparative Expression Profiling in Pulmonary Fibrosis Suggests a Role of Hypoxia-Inducible Factor-1α in Disease Pathogenesis. Am. J. Respir. Crit. Care Med..

[13] Zhang W., Shi X., Peng Y., Wu M., Zhang P., Xie R., Wu Y., Yan Q., Liu S., Wang J. (2015). HIF-1α Promotes Epithelial-Mesenchymal Transition and Metastasis through Direct Regulation of ZEB1 in Colorectal Cancer. PLoS ONE.

[14] Mingyuan X., Qianqian P., Shengquan X., Chenyi Y., Rui L., Yichen S., Jinghong X. (2017). Hypoxia-Inducible Factor-1α Activates Transforming Growth Factor-B1/Smad Signaling and Increases Collagen Deposition in Dermal Fibroblasts. Oncotarget.

[15] Gusarova G.A., Trejo H.E., Dada L.A., Briva A., Welch L.C., Hamanaka R.B., Mutlu G.M., Chandel N.S., Prakriya M., Sznajder J.I. (2011). Hypoxia Leads to Na,K-ATPase Downregulation via Ca2+ Release-Activated Ca2+ Channels and AMPK Activation. Mol. Cell. Biol..

[16] Davis F.M., Azimi I., Faville R.A., Peters A.A., Jalink K., Putney J.W., Goodhill G.J., Thompson E.W., Roberts-Thomson S.J., Monteith G.R. (2014). Induction of Epithelial–Mesenchymal Transition (EMT) in Breast Cancer Cells Is Calcium Signal Dependent. Oncogene.

[17] Zhong Q., Zhou B., Ann D.K., Minoo P., Liu Y., Banfalvi A., Krishnaveni M.S., Dubourd M., Demaio L., Willis B.C. (2011). Role of Endoplasmic Reticulum Stress in Epithelial–Mesenchymal Transition of Alveolar Epithelial Cells: Effects of Misfolded Surfactant Protein. Am. J. Respir. Cell Mol. Biol..

[18] Uzunhan Y., Bernard O., Marchant D., Dard N., Vanneaux V., Larghero J., Gille T., Clerici C., Valeyre D., Nunes H. (2016). Mesenchymal Stem Cells Protect from Hypoxia-Induced Alveolar Epithelial-Mesenchymal Transition. Am. J. Physiol. Lung Cell. Mol. Physiol..

[19] Williams J.A., Hou Y., Ni H.-M., Ding W.-X. (2013). Role of Intracellular Calcium in Proteasome Inhibitor-Induced Endoplasmic Reticulum Stress, Autophagy and Cell Death. Pharm. Res..

[20] Planes C., Friedlander G., Loiseau A., Amiel C., Clerici C. (1996). Inhibition of Na-K-ATPase Activity after Prolonged Hypoxia in an Alveolar Epithelial Cell Line. Am. J. Physiol. Lung Cell. Mol. Physiol..

[21] Zhou G., Dada L.A., Wu M., Kelly A., Trejo H., Zhou Q., Varga J., Sznajder J.I. (2009). Hypoxia-Induced Alveolar Epithelial-Mesenchymal Transition Requires Mitochondrial ROS and Hypoxia-Inducible Factor 1. Am. J. Physiol. Lung Cell. Mol. Physiol..

[22] Tanjore H., Xu X.C., Polosukhin V.V., Degryse A.L., Li B., Han W., Sherrill T.P., Plieth D., Neilson E.G., Blackwell T.S. (2009). Contribution of Epithelial-Derived Fibroblasts to Bleomycin-Induced Lung Fibrosis. Am. J. Respir. Crit. Care Med..

[23] Chen X., Iliopoulos D., Zhang Q., Tang Q., Greenblatt M.B., Hatziapostolou M., Lim E., Tam W.L., Ni M., Chen Y. (2014). XBP1 Promotes Triple-Negative Breast Cancer by Controlling the HIF1α Pathway. Nature.

[24] Li H., Chen X., Gao Y., Wu J., Zeng F., Song F. (2015). XBP1 Induces Snail Expression to Promote Epithelial- to-Mesenchymal Transition and Invasion of Breast Cancer Cells. Cell. Signal..

[25] Banerjee A., Ahmed H., Yang P., Czinn S.J., Blanchard T.G. (2016). Endoplasmic Reticulum Stress and IRE-1 Signaling Cause Apoptosis in Colon Cancer Cells in Response to Andrographolide Treatment. Oncotarget.

[26] Mo X.-T., Zhou W.-C., Cui W.-H., Li D.-L., Li L.-C., Xu L., Zhao P., Gao J. (2015). Inositol-Requiring Protein 1 —X-Box-Binding Protein 1 Pathway Promotes Epithelial–Mesenchymal Transition via Mediating Snail Expression in Pulmonary Fibrosis. Int. J. Biochem. Cell Biol..

[27] Bhattacharya A., Kumar J., Hermanson K., Sun Y., Qureshi H., Perley D., Scheidegger A., Singh B.B., Dhasarathy A. (2018). The Calcium Channel Proteins ORAI3 and STIM1 Mediate TGF-& β2; Induced *Snai1* Expression. Oncotarget.

[28] Krebs J., Agellon L.B., Michalak M. (2015). Ca2+ Homeostasis and Endoplasmic Reticulum (ER) Stress: An Integrated View of Calcium Signaling. Biochem. Biophys. Res. Commun..

[29] Belaidi E., Thomas A., Bourdier G., Moulin S., Lemarié E., Levy P., Pépin J.-L., Korichneva I., Godin-Ribuot D., Arnaud C. (2016). Endoplasmic Reticulum Stress as a Novel Inducer of Hypoxia Inducible Factor-1 Activity: Its Role in the Susceptibility to Myocardial Ischemia-Reperfusion Induced by Chronic Intermittent Hypoxia. Int. J. Cardiol..

[30] Bernard O., Jeny F., Uzunhan Y., Dondi E., Terfous R., Label R., Sutton A., Larghero J., Vanneaux V., Nunes H. (2018). Mesenchymal Stem Cells Reduce Hypoxia-Induced Apoptosis in Alveolar Epithelial Cells by Modulating HIF and ROS Hypoxic Signaling. Am. J. Physiol. Lung Cell. Mol. Physiol..

[31] Migneault F., Boncoeur É., Morneau F., Pascariu M., Dagenais A., Berthiaume Y. (2013). Cycloheximide and Lipopolysaccharide Downregulate ΑENaC MRNA via Different Mechanisms in Alveolar Epithelial Cells. Am. J. Physiol. Lung Cell. Mol. Physiol..

[32] Chaveroux C., Sarcinelli C., Barbet V., Belfeki S., Barthelaix A., Ferraro-Peyret C., Lebecque S., Renno T., Bruhat A., Fafournoux P. (2016). Nutrient Shortage Triggers the Hexosamine Biosynthetic Pathway via the GCN2-ATF4 Signalling Pathway. Sci. Rep..

