# Supplementary material for: ER Stress is Involved in Epithelial-To-Mesenchymal Transition of Alveolar Epithelial Cells Exposed to a Hypoxic Microenvironment
Source: Int J Mol Sci. 2019 Mar 14;20(6):1299. doi: 10.3390/ijms20061299 (PMC6470993; doi:10.3390/ijms20061299)
Supplement: Supplementary file 1 [file ijms-20-01299-s001.pdf]

SUPPLEMENTAL DATA

ROS production measurement. 30 mg of snap-frozen rat lung biopsies exposed several time (16 h-48 h) to hypobaric hypoxia or normoxia were washed twice in PBS and then subjected to an Ultra-Turrax® homogenizer (IKA) in protein lysis buffer containing 20 mM Tris base, 150 mM NaCl, 1% Triton X100, 1% SDS, 0,5% deoxycholate and 0.01% cocktail protease inhibitors (Thermo Scientific). Samples of 40 µg proteins were incubated with 10 µM 2',7'-dichlorodihydrofluorescein diacetate (H_2DCFDA) (Molecular Probes) for 90 min at 37°C in the dark. The presence of oxidative stress was evaluated by the conversion of non-fluorescent 2,7-dichlorofluorescein diacetate (H_2DCFDA) to fluorescent 2,7-dichlorofluorescein (DCF) by ROS present within the cells. Fluorescence was measured by a TriStar2 LB942 microplate reader (Berthold Technologies). Values of fluorescence intensity were reported.

AOPP level quantification. Lung proteins were extracted from rat exposed several time (16 h-48 h) to hypobaric hypoxia or normoxia. Samples of 10 µg proteins were incubated in a 96 well plates with the chloramine reaction initiator (CellBiolabs). After 5 min, the reaction was stop and the absorbance was read at 340 nm. The concentration of advanced oxidized protein products was reported (mmol/L).

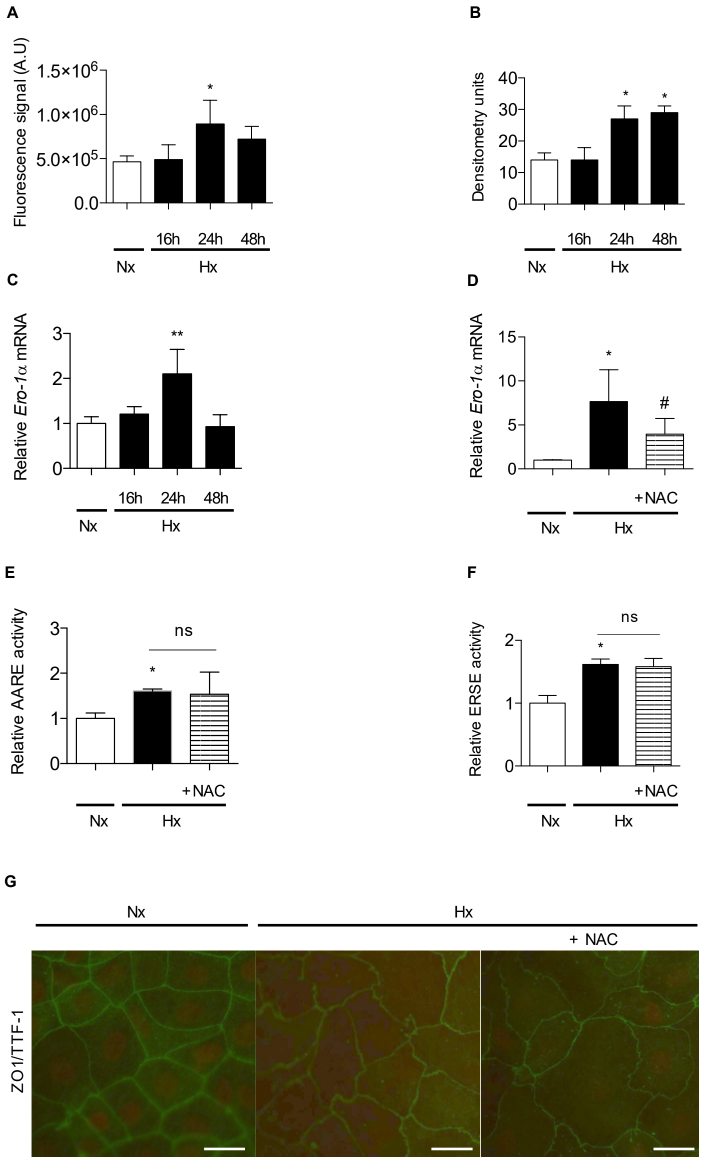

Supplemental figure 1. Oxidative unbalance in ER is not critical for UPR pathways activation in hypoxic AECs. (A-C) Lungs of rats stabulated in normoxia (Nx) (21 % O_2) or exposed to hypoxia (Hx) (8 % FiO_2-like) during 16 h, 24 h or 48 h were isolated and used for oxidative state measurements and RT-qPCR analyses. ROS level (A) and advanced oxidation protein products and Ros production (B) were evaluated in lung homogenates. (C) mRNA transcript expression levels of *Ero-1α* in lungs homogenates of rats exposed 16 h, 24 h or 48 h to hypoxia were quantified by qRT-PCR using $2^{-\Delta\Delta CT}$ method and reported to the normoxic condition. n= 5 rats for each group. (D-G) Isolated primary rat AECs were cultured in normoxia (Nx) (21 % O_2) or hypoxia (Hx) (1.5% O_2) during various periods in the presence or absence of 5 mM N-acetyl-L-cystein (NAC). (D) mRNA expression levels of *Ero-1α* were quantified by qRT-PCR using $2^{-\Delta\Delta CT}$ method in rat AECs cultured in the presence or absence of 5 mM NAC and exposed 6 h to normoxia or hypoxia. mRNA levels under hypoxic condition were reported to the normoxic condition. (E) Primary rat AECs transfected with plasmid coding for luciferase reporter activity of amino acid response element (AARE: *ie* ATF4-luc) or (F) endoplasmic reticulum stress element (ERSE: *ie* ATF6/XBP-1-luc), were treated or not with 5 mM NAC and exposed 6 h in hypoxic condition. Luciferase activity corresponding to the transcriptional capacity of ATF4 (E) or (F) ATF6N was measured. n = 4 experiments were performed. Raw data were submitted to a Mann-Whitney test or a Kruskal-Wallis one-way analysis of variance. * and ** indicate a significant difference as compared with normoxic condition with a $P<0.05$ and $P<0.01$, respectively. ns indicate a non-significant difference as compared with hypoxic condition. (G) ZO-1 (green) and TTF1 (red) immunostaining were performed on rat AECs cultured on filter and exposed for 6-days to hypoxia in the presence or absence of 5 mM NAC. A representative picture of at least n=4 independent experiment for each condition has been presented.
